# The Function of the Autonomic Nervous System in Asian Patients With Chronic Migraine

**DOI:** 10.3389/fnins.2022.773321

**Published:** 2022-04-14

**Authors:** Min Shi, Danqing Luo, Jun Guo, Dongdong Yang, Zhaoying Li, Huan Zhao

**Affiliations:** ^1^Department of Neurology, Hospital of Chengdu University of Traditional Chinese Medicine, Chengdu, China; ^2^Department of Rehabilitation, Hospital of Chengdu University of Traditional Chinese Medicine, Chengdu, China

**Keywords:** chronic migraine, autonomic nervous system, cold pressor test, pupillary light reflex, functional nuclear magnetic resonance imaging

## Abstract

**Background:**

The pathogenic mechanisms underlying the autonomic nervous system (ANS) dysfunction in patients with chronic migraine (CM) remain unclear. This study investigated the pathogenesis of ANS dysfunction in this population.

**Methods:**

A total of 60 patients diagnosed with CM and 60 healthy subjects were recruited to participate in this study. The pupil diameter, pupil contraction velocity, latency, amplitude, and the maximum gradient recovery time were examined before, at 2 min and at 5 min after the cold pressor test, which was combined with the pupillary light reflex method. A brain 3D T1-weighted structural imaging scan, resting-state functional magnetic resonance imaging scan, and diffusion tensor imaging (DTI) scan were also acquired.

**Results:**

Patients with CM exhibited a longer recovery time to the maximum gradient at 2 min and at 5 min after cold pressing compared with the control group (*P* < 0.01 and *P* < 0.05, respectively). There was no significant difference in the pupil diameter, pupillary contraction velocity, latency, amplitude, blood pressure, or heart rate between the two groups (all *P* > 0.05). In the CM group, the regional homogeneity (ReHo) values of the left amygdala and left lateral hypothalamic area were significantly higher than those of other brain areas (*P* < 0.001, Alphasim corrected). The DTI scan of the whole brain area showed a lack of significant difference in DTI indices, including FA, MD, AD, and RD values between the two groups (*P* > 0.05, Alphasim corrected).

**Conclusion:**

The dysfunction of the left amygdala and left lateral hypothalamic area may be related to ANS dysfunction in patients with CM.

## Introduction

Migraine is one of the oldest documented human diseases, dating back to ancient Egyptian times (∼1200 BC) (Miglis, 2018). It is a common recurrent primary headache that most affects people aged 15–49 years old (Steiner et al., 2018). Chronic migraine (CM) is defined as the persistence of headaches of any duration or severity for ≥15 days and for ≥8 days that meet specific migraine criteria and which seriously affects the quality-of-life of patients (Riesco et al., 2016). Approximately 82% of patients with CM experience at least one parasympathetic symptom such as lacrimation, conjunctival congestion, eyelid edema, ear distension, and nasal congestion, indicating a dysfunction of the autonomic nervous system (ANS) (Straube et al., 2015). Premonitory symptoms, such as nausea and vomiting, often start before the onset of attacks in patients with migraine with aura, which is also a manifestation of ANS dysregulation. In addition, previous evidence has suggested that regulating parasympathetic function may be effective in preventing or terminating migraine attacks (Eren et al., 2018). Although progress has been made in the treatment of migraine, the pathogenic mechanisms, especially regarding the activation of the ANS, remain elusive. Previous studies have investigated the function of the ANS in patients with episodic migraine (EM), but the results are inconsistent and at times contradictory (Mylius et al., 2003; Bugdayci et al., 2010; Torun et al., 2013; Cambron et al., 2014; Cernuda-Morollón et al., 2015; Matei et al., 2015; Mamontov et al., 2016; Eren et al., 2018). Few studies have focused on the ANS function of patients with CM.

Many methods have been used to assess the ANS function of patients with migraine, including heart rate variability analysis (Bugdayci et al., 2010), pupillary light reflex (Mylius et al., 2003; Matei et al., 2015; Mamontov et al., 2016), analysis of salivary α-amylase level (Cambron et al., 2014), and the detection of vasoactive intestinal peptide (VIP) (Torun et al., 2013). The cold pressor test (CPT) is an effective method used to induce systemic sympathetic activation (Eren et al., 2018). The combination of the CPT and the pupillary light reflex has yet to be used to assess the ANS function of patients with CM. The pupillary light reflex method is a mature (Mylius et al., 2003; Cambron et al., 2014; Eren et al., 2018), feasible, widely used (Giza et al., 2011; Yuan et al., 2014; Koike et al., 2016; Chougule et al., 2019; Bower et al., 2021), and is a non-invasive method for the assessment of the ANS function. The advantages of this method include the simultaneous assessment of the sympathetic and parasympathetic nervous system, few interfering factors, simultaneous measurement of heart rate and blood pressure, and an assessment of cardiac innervation.

Functional magnetic resonance imaging (fMRI) and diffusion tensor imaging (DTI) are mainly used in imaging studies of chronic migraine (Lu et al., 2019). Resting-state fMRI (rs-fMRI) examination has revealed abnormalities in brain areas related to pain processing in patients with CM, including the sensorimotor network, prominent network, executive control network, default network, frontal parietal network, midbrain periaqueductal gray matter network, etc. Functional impairments in the sensory, visual, and cognitive brain areas have also been observed in these patients. However, most of these studies had a small sample size and focused on patients with recurrent migraine. Therefore, little is known regarding patients with CM. In this study, fMRI and CPT combined with the pupillary light reflex method were performed to investigate the pathological mechanisms of ANS dysfunction in patients with CM.

## Materials and Methods

### Subjects

Sixty patients diagnosed with CM at the Department of Neurology, Affiliated Hospital of Chengdu University of TCM between April 2019 and April 2020 were recruited to participate in the current study. The inclusion criteria were as follows: (1) diagnosed with CM according to the Diagnostic Criteria of the ICHD-III CM issued by the International Headache Society in 2018 (IHS, 2018); (2) cranial MRI and common electrocardiogram examination showed no signs of diseases of other systems; (3) had no eye diseases or history of eye surgery; (4) aged between 18 and 55 years; (5) had no headache attack 3 days before, on the day, or 1 day after the fMRI scan; (6) had no other mental or psychological conditions; (7) had no history of drug abuse; (8) the result of nervous system examination was normal. Acute symptomatic treatment during the attack period was allowed. The exclusion criteria included: (1) had a history of autonomic dysfunction, such as syncope or postural tachycardia syndrome; (2) diagnosed with aural vertigo, cervical vertigo, vertigo epilepsy, multiple sclerosis, stroke, cardiovascular disease, diabetes, obesity, or other metabolic disorders; (3) had a history of infection 1 month before enrollment; (4) took painkillers within 2 weeks of enrollment or took vitamin B within 1 month of enrollment; (4) had other organic diseases or tumors; (5) was on a diet, suffered from malnutrition, or was pregnant; (6) had corneal, conjunctival, or pupillary lesions, or other ocular discomfort; (7) had chronic pain and a history of long-term sleep disorders; (8) had claustrophobia; (9) had metal dentures, cardiac stents, pacemakers, artificial joints, or other magnetic resonance contraindications; (10) had drug abuse-related CM according to the Silberstein criteria, including the abuse of NSAIDs (>15 days/month), triptan (>10 days/month), NSAIDs and triptan (NSAIDS: >15 days/month; triptan: >10 days/month), ergotamine, caffeine, and opioid/barbiturate (single or combined use with NSAIDs or triptan; >10 days/month). The frequency of acute analgesics taken by patients with CM is shown in Table 1.

**TABLE 1:** The frequency of acute analgesics taken by patients with CM (days/month).

Drug	Frequency
NSAIDs	6.12 ± 1.23
Triptan	4.06 ± 0.78
Ergotamine	2.34 ± 1.86
Caffeine	5.56 ± 2.89
Opioid/barbiturate	3.34 ± 1.22

The disease course was 3–10 years in 21 patients, 10–20 years in 21 patients, and >20 years in 8 patients. The average visual analogue scale (VAS) score of all patients was 5–9 (moderate and severe pain). Fifty-seven patients had attacks accompanied by nausea, vomiting, and/or photophobia. Thirty patients had a family history of migraine. Fifty-seven patients did not take preventive medications regularly for 1 month. Two patients stopped taking preventive medications for over 7 months.

A healthy control (HC) group consisting of 60 healthy subjects was also enrolled, including 51 females and 9 males. The inclusion criteria were as follows: (1) aged between 20 and 55 years; (2) had no history of migraine or other chronic primary headaches; (3) had no cardiovascular and neurological diseases; (4) had no eye diseases or history of eye surgery; (5) exhibited normal results in a physical examination within the past 6 months. The sociodemographic characteristics of patients with CM and the HC group are shown in Table 2.

**TABLE 2:** Sociodemographic characteristics of patients with CM and healthy controls.

Characteristics/categories	CM group (*n* = 60)	HC group (*n* = 60)
**Age (years)**		
18–29	38.3	31.7
30–41	40.7	40.0
42–55	20.0	29.3
**Sex**		
Men	30	15
Women	70	85
**Race**		
Asia	100	100
**Education**		
≤High school	20	15
College	45	38.3
Graduate school	35	46.7
**Smoking status**		
Never	50	66.7
Past	30	18.3
Current	20	15
**Alcohol use, servings/week**		
None	70	81.67
1–7	20	13.33
8+	10	0.05
**Use of oral contraceptives**		
Never	53.33	65.00
Past	35.00	28.33
Current	11.64	6.67

*Data are shown as percentages (%).*

All subjects were informed of the purpose, content, withdrawal rights, and confidentiality principles of the study before enrollment. This study was approved by the Medical Ethics Committee of the Affiliated Hospital of Chengdu University of TCM (2019KL-061). All patients provided written informed consent form and the clinical trial registration was completed in China (registration number: chiCTR1900028542).

### Pupillometry

All subjects were asked not to take benzodiazepines or drink alcohol or coffee within 24 h before the test. Female subjects were tested during the non-menstrual period. Patients with CM underwent pupillometry during the remission period (at least 48 h apart from the most recent migraine attack and without any headache symptoms or other discomfort symptoms at the time of test). The binocular pupil analyzer used in this study was developed by the Suzhou Institute of Biomedical Engineering and Technology, Chinese Academy of Sciences. It is a closed box with a built-in binocular infrared camera and a binocular stimulation light source [power: 0.6 W, wavelength: 525 nm, voltage: 3.0∼3.7 V (10 series), current: 20 mA, brightness: 100 Lm]. The test was performed in a room with a temperature of 25°C. The subject was seated upright with both eyes placed above the closed eyepiece, which were both observed through a computer monitor. The pupil was located in the center of the window. After darkness adaptation for 10 min, the assessment started. The subject was instructed to open his/her eyes as much as possible and to not blink or rotate his/her eyeballs during the assessment.

### Cold Pressor Test

**FIGURE 1:**
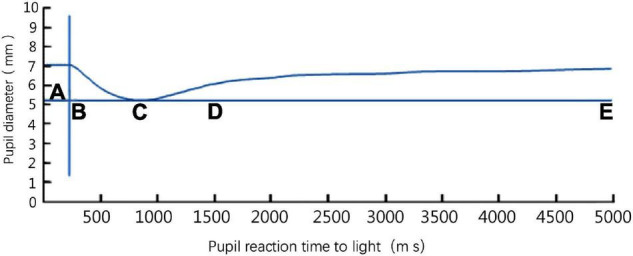
The pupil light response curve of a left eye detected by a binocular pupil analyzer. Phase AB: latency of pupillary light reflex; phase BC: period of pupillary light reflex contraction; phase CE: recovery phase (dilation period); D: time to maximum gradient recovery; phase CD: the first phase of recovery; phase DE: the second phase of recovery.

The subject was instructed to place his/her left hand in water at 4°C, with the wrist submerged and five fingers extended for 5 min. The parameters of the pupillary light reflex were measured before, and at 2 min and 5 min after cold pressing. Each measurement lasted 5 s. The parameters of the left eye were continuously measured three times at each time point, and the mean value was used for analysis. The parameters of the pupillary light reflex included (1) pupil diameter: the diameter of the pupil before light stimulation (mm), (2) pupil contraction velocity: the mean velocity of the pupil contraction to the minimum diameter (mm/s); (3) latency: the time interval between light stimulation and the start of contraction (ms); (4) amplitude: the difference between the initial diameter and the minimum pupil diameter (mm); (5) time to maximum gradient recovery: the time when the pupil dilation velocity reached the peak at the initial stage of recovery (i.e., the time corresponding to the maximum slope of the pupil recovery curve on the pupil light response curve) (Figure 1). Blood pressure and heart rate were measured at each time point using an arm sphygmomanometer.

### Magnetic Resonance Imaging Acquisition

Functional MRI images of patients with CM were obtained at ≥24 h after the last attack. High-resolution 3D T1-weighted and diffusion-weighted MRI data were collected with a 32-channel coil and a GE 3.0 T magnetic resonance imager (GE DISCOVERY MR750, General Electric Company, Fairfield, United States). All images were axial scans. The field-of-view (FOV) ranged from the top of the head to the lower edge of the cerebellar tonsils, with the AC-PC line as the reference plane.

The brain 3D T1-weighted structural image were obtained using a gradient echo sequence 3D-FSPGR with the following parameters: repetition time (TR)/echo time (TE) = 8.208 ms/3.22 ms, flip angle (FA) = 12, spacing between slices (SBS) = 1 mm, interslice distance = 0.5 mm, FOV = 240 mm × 240 mm, matrix = 256×256, number of slices = 40. A total of 312 slices were collected with a scanning time of 4 min and 45 s.

An echo-planar imaging (EPI) sequence and axial scanning were adopted for a resting-state (rs)-fMRI scan with the following parameters: TR/TE = 2000 ms/30 ms, FOV = 240 mm × 240 mm, matrix = 64×64, FA = 90, SBS = 4 mm, interslice distance = 4 mm/1 mm, voxel size = 3.75 mm × 3.75 mm × 4 mm, number of slices = 40. A total of 240 time points were collected with a scanning time of 7 min.

An EPI sequence and axial scanning were adopted for a diffusion tensor imaging (DTI) scan with the following parameters: TE = 88.6 ms, TR = 6800 ms; SBS = 3 mm, interslice distance = 0, FA = 90, 25 diffusion directions, *b* value = 1000 s/mm. A total of 1,256 slices were collected with a scanning time of 6 min and 14 s.

### Image Processing

The DPABI-Data Processing Assistant for Resting-State fMRI V4.4 software was used for data analysis. DICOM data were initially converted to the NIfTI format, the first 10 time points were removed, and slice-timing correction was applied. Data with >1 mm translation and/or >1° rotation were removed after head movement correction. The structural image and functional image were aligned, and the functional data were then resampled to a voxel size of 3 mm × 3 mm × 3 mm. The structural and functional data were then warped to a standard coordinate space. A Gaussian kernel of 6 mm × 6 mm × 6 mm was applied for spatial smoothing. The functional data was then bandpass filtered between 0.01 and 0.08 Hz to remove linear drift. The ReHo values were then calculated. The Kendall’s coefficient concordance (also known as Kendall’s W) at each voxel was calculated using a predefined neighborhood. A sphere with a radius of 6 mm was used. The Kendall’s W ranges from 0 to 1, where 0 means no coherence and 1 means absolute coherence. The ReHo map was generated using all voxels’ Kendall’s W.

The 19 ANS-related regions were identified by Benarroch (1993) and Borsook et al. (2012). The Pick atlas was used to identify 19 seed regions of interest (ROI, voxels within a 6-mm radius sphere) in the periaqueductal gray matter, parabrachial, dorsal nucleus of the vagus nerve (DMV), and ventrolateral medulla: left-amygdala, right-amygdala, left-parauentricular, right-parauentricular, left-parauentricular, right-parauentricular, left-insular cortex, right-insular cortex, left-parabrachial region, right-parabrachial region, left-nucleus of the tractus solitarius, right-nucleus of the tractus solitarius, left-nucleus ambiguus, right-nucleus ambiguus, left-ventrolateral medulla, right-ventrolateral medulla, left-lateral hypothalamic area, right-lateral hypothalamic area, and periaqueductal gray matter (Maldjian et al., 2003). The signal time series of each ROI was extracted from the spatially normalized resting-state image of each subject.

A Linux virtual machine was created using the VM VirtualBox.^1^ The FSL software (version 5.0) was used to remove non-brain voxels in the DTI scans belonging to the skull. Eddy head correction was also performed. The FA value was calculated and registered to the standard space and were then smoothed.

The FreeSurfer software (stable version 6.0) was used to process the 3D-T1-weighted sequence images. The image data were automatically processed using the “recon-all-qcache” command to obtain the cortical thickness data of each area of the brain and cerebellum. Then, the data were extracted using the MATLABR2012b software. The FreeSurfer software (development version) was also used to perform the “segmentHA_T1.sh” command for processed data files. This command was used to segment the amygdala, hippocampus, and brainstem.

The VBM8 software (based on the MATLAB R2012b platform) was used to process the data of 3D-T1-weighted sequence images to determine whether they were consistent with the results obtained by FreeSurfer.

Conventional T1-weighted MRI volumes were processed before statistical analysis by Tract based spatial statistics (TBSS) (Smith et al., 2006). Four DTI indices were obtained, including the FA, representing the directivity of water diffusivity, the mean diffusivity (MD), indicating the overall measure of water diffusion, the radial diffusivity (RD), reflecting diffusivity perpendicular to the principal direction, and the axial diffusivity (AD). The FA, MD, RD, and AD values indicate diffusivity of white matter fibers in the principal direction (Pelletier et al., 2015).

### Statistical Analysis

Statistical analysis was performed using the SPSS 24.1 software. The Kolmogorov–Smirnov and Levene’s test for equality of variances were used to assess the normality and homogeneity of the variance of age and migraine duration (years). If the null hypothesis in the Kolmogorov–Smirnov and Levene’s test was not rejected, a one-way analysis of variance was used to compare the data between the two groups. Otherwise, the Kruskal–Wallis test was used. A Chi-square test was used to determine statistical significance in gender between the two groups. A permutation-based reasoning tool was used to determine the voxel-based TBSS difference of the FA, MD, AD, and RD values of white matter between the two groups using a non-parametric statistical method called “randomization.” This tool is implemented in the FSL software and has the threshold-free cluster enhancement option (Nichols and Holmes, 2002; Smith and Nichols, 2009).

Five thousand permutations were chosen to allow robust statistical inference, and the significance threshold of the difference between groups was *P* < 0.05 after applying the threshold-free cluster enhancement option. When significant differences were detected, additional clinical covariates were added for comparison. These covariates were analyzed separately to assess the effect of each covariate. *P* < 0.05 was considered statistical significance.

The ReHo values of the brain regions that were statistically different between the CM and HC groups were extracted using the ROI Signal Extractor in the DPABI software. A two-sample *t*-test was used to determine the cluster MMSE and frame displacement regression with ReHo differences between CH patients and HC. Multiple comparisons were corrected for by AlphaSim. The minimum cluster size calculated by the AlphaSim program embedded in DPABI and a voxel threshold of *P* < 0.001 were used to achieve the correction as determined by Monte Carlo simulation with *P* < 0.05.^2^ The cut-off cluster size determined by AlphaSim ranged between 154 mm^3^ (with CM cases alone) and 217 mm^3^ (with all participants) voxels. The resulting statistical map was set with a combined threshold of *P* < 0.05, a minimum cluster size of 217 mm^3^, corresponding to a corrected threshold of *P* < 0.05. The generated t-map was superimposed on the axial view in the rendering view and slice using BrainNet Viewer, which embeds the DPABI viewer module.^3^ The anatomical structure of the brain region was visualized by the xjView software.^4^

## Results

### Demographic and Clinical Features of the Chronic Migraine and Healthy Control Groups

A total of 60 patients with CM and 60 healthy subjects were recruited according to the previously described inclusion and exclusion criteria. Conventional MRI showed that there was no structural abnormality in either group. There were three dropouts (one in the HC group and two in the CM group) due to errors caused by the application of non-linear registration to the FMRIB-58 images and poor patient compliance. The demographic and clinical characteristics of the two groups are shown in Table 3.

**TABLE 3:** Demographic and clinical characteristics of the CM and HC groups.

	CM group (*n* = 60)	HC group (*n* = 60)	Statistics	*P*-value
Age (Year)	34.7 ± 7.3	36.7 ± 6.5	*F* = 0.333	0.718
Gender Male/Female	42/18	51/9	χ^2^ = 0.784	0.882
Headache frequency (times/month)	18 ± 3.6			
Duration of headache (h/time)	6.80 ± 2.7			
Disease course (Year)	17.4 ± 8.0			
VAS (score)	8.1 ± 1.5			

### Pupillary Light Reflex Parameters of the Chronic Migraine and Healthy Control Groups

**FIGURE 2:**
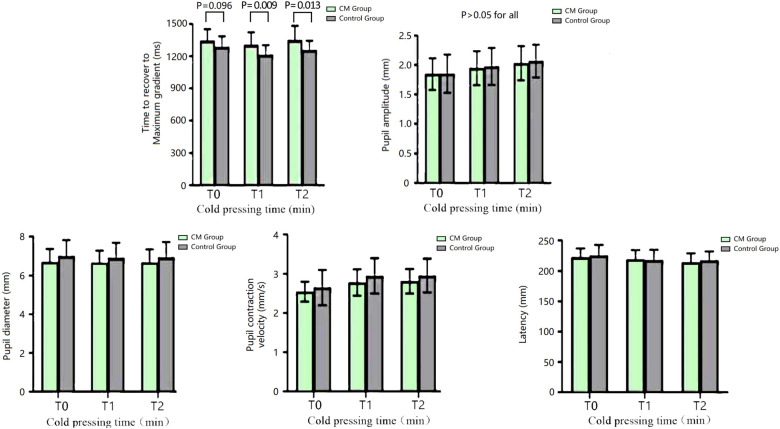
The parameters of the pupillary light reflex of the CM and HC groups at different time points of the CPT. CM, chronic migraine.

The time to maximum gradient recovery before cold pressing did not significantly differ between the two groups (*P* > 0.05, Alphasim corrected). Patients with CM exhibited a significantly longer time to maximum gradient recovery at both 2 min and 5 min after cold pressing compared with the HC group (*P* < 0.01 and *P* < 0.05, respectively, Alphasim corrected). There was no significant difference in the amplitude, pupil diameter, contraction velocity, and latency of pupillary light reflex between the two groups at all time points (*P* > 0.05, Alphasim corrected) (Figure 2).

### Heart Rate and Blood Pressure of the Chronic Migraine and Healthy Control Groups

**FIGURE 3:**
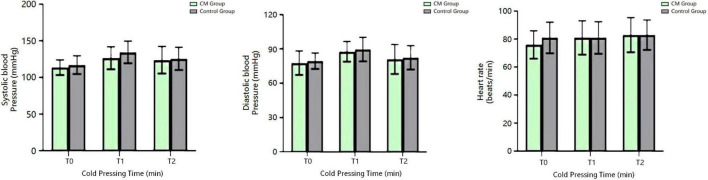
Blood pressure and heart rate of the CM and HC groups at different time points of the CPT. CM, chronic migraine.

There was no significant difference in the heart rate and blood pressure between the two groups at all time points of the CPT (*P* > 0.05, Alphasim corrected) (Figure 3).

### Resting-State Functional Magnetic Resonance Imaging and Regional Homogeneity of the Chronic Migraine and Healthy Control Groups

**FIGURE 4:**
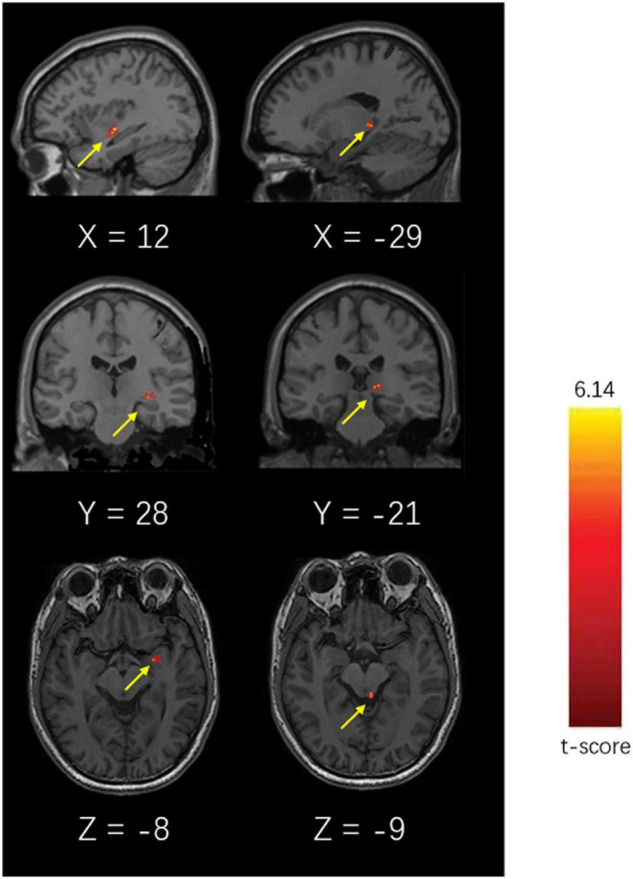
Brain diagram of different ReHo values of the CM and HC groups (*P* < 0.05, Alphasim Alphasim corrected).

The ReHo values of the left amygdala and left lateral hypothalamic area of patients with CM were significantly higher than those of the HC group (*P* < 0.05, Alphasim corrected). The highest *T*-value was located in the left amygdala and the left lateral hypothalamic area (Table 4 and Figure 4).

**TABLE 4:** Brodmann area of peak voxel with altered ReHo values in rs-fMRI.

Area of peak voxel	Brodmann area	Cluster size (voxels)	Coordinates	*T*-value	*P*-value
Left amygdala	32	300	X = 12 Y = 28 Z = −8	5.13	<0.01
Left lateral hypothalamic area	21	773	X = −29 Y = −21 Z = −9	6.14	<0.01

### Tract Based Spatial Statistics Analysis of White Matter Skeleton *via* a Pipeline Toolbox for Analyzing Brain Diffusion Images

**FIGURE 5:**
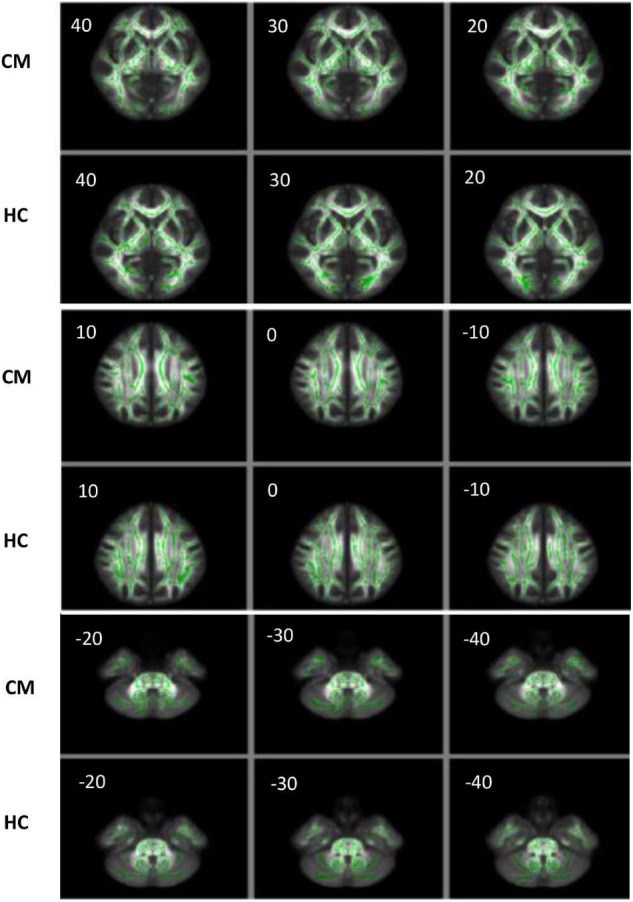
The FA image of the CM and HC groups analyzed by TBSS. The images of the two groups were visualized using the FSLview software, and the FA values were analyzed by TBSS. The green areas are the average FA skeleton of the white matter. The red and yellow areas represent voxels that indicate changes in the DTI value related to the white matter. A threshold of *t* = 0.2 was used (*P* < 0.05). The closer the voxel color is to yellow, the smaller the *P*-value. The closer the voxel color is to red, the closer the *P*-value to 0.05, which indicates more changes in the FA value of the corresponding brain area. When *P* > 0.05, only green and white matter skeletons are shown.

The output results showed that there was no significant difference in the FA, AD, RD, and MD of 19 ANS-related brain areas between the CM and HC groups (*P* > 0.05) (Figure 5 and Table 5).

**TABLE 5:** The *P*-values of the FA, MD, AD, and RD values of 19 ANS-related brain regions between the CM and HC groups.

Brain area	FA (*P*-value)	MD (*P*-value)	AD (*P*-value)	RD (*P*-value)
Left amygdala	0.240	0.457	0.267	0.128
Right amygdala	0.345	0.223	0.765	0.345
Periaqueductal gray matter	0.567	0.368	0.213	0.674
Left paraventricular	0.078	0.234	0.113	0.078
Right paraventricular	0.082	0.024	0.345	0.512
Left-paraventricular	0.147	0.113	0.488	0.145
Right paraventricular	0.346	0.197	0.411	0.083
Left insular cortex	0.562	0.336	0.442	0.067
Right insular cortex	0.109	0.721	0.095	0.873
Left parabrachial region	0.056	0.076	0.108	0.562
Right parabrachial region	0.389	0.625	0.077	0.065
Left nucleus of the tractus solitarius	0.445	0.321	0.655	0.082
Right nucleus of the tractus solitarius	0.089	0.056	0.178	0.211
Left nucleus ambiguus	0.881	0.092	0.077	0.321
Right nucleus ambiguus	0.367	0.134	0.512	0.276
Left ventrolateral medulla	0.452	0.098	0.671	0.197
Right ventrolateral medulla	0.167	0.256	0.561	0.045
Left lateral hypothalamic area	0.089	0.113	0.375	0.071
Right lateral hypothalamic area	0.112	0.059	0.078	0.773

### Adverse Events of the Chronic Migraine and Healthy Control Groups

One female patient in the CM group experienced syncope at 5 min after cold pressing and woke up spontaneously after 2 min. She was discharged after 30 min of observation in the hospital without signs of discomfort. This patient was excluded from subsequent analysis. Eight patients in the CM group experienced migraine within 24 h of the test, with a VAS score of 2–3. Two patients were relieved after taking non-steroidal analgesics. The remaining six patients had headaches for about 3 h and were relieved spontaneously without medications. No adverse events were observed in the HC group.

## Discussion

The ANS is composed of sympathetic and parasympathetic nerves, which maintain a dynamic balance to ensure normal functioning of the body. The size of the pupil depends on many factors (e.g., light, environment, emotional state, etc.) and often reflects the balance between sympathetic and parasympathetic tension. The pupillary light reflex is regulated by a parasympathetically innervated pupillary sphincter and a sympathetically innervated pupillary dilator muscle (Giza et al., 2011). Pupillary light reflex latency, contraction velocity, time to maximum gradient recovery at the initial recovery phase, dilation time (velocity) at the initial recovery phase, and pupillary amplitude are controlled by parasympathetic nerves. By contrast, pupil diameter and dilation time (velocity) at the second stage of recovery are driven by sympathetic nerves. When a light stimulus is removed, the pupil diameter returns to the baseline level. This process can be divided into two stages. The pupillary dilation velocity at the initial stage is fast, in which the active parasympathetic nerve drives the pupillary sphincter to withdraw to control the pupil. The pupillary dilation velocity at the second stage is relatively slow, a process by which the sympathetic nerve activity dominates and drives the pupillary dilator muscle to slowly restore the pupil to the baseline diameter (Lu et al., 2019). Thus, the initial stage of the recovery phase is predominately innervated by parasympathetic nerves, while the second stage is mainly innervated by sympathetic nerves.

Eren et al. (2018) found that there was a slight delay in the time to achieve the maximum dilation velocity at the initial stage of pupillary recovery in patients with EM. Furthermore, the dilation velocity did not reach the maximum until 5 min after cold pressing, while the dilation velocity of healthy subjects reached the maximum at 2 min after cold pressing. In addition, the pupillary contraction velocity of patients with EM was significantly higher than that of the control group at 5 min after cold pressing, suggesting that patients with EM may have parasympathetic dysfunction. Cernuda-Morollón et al. (2015) found that the VIP level of patients with CM (*n* = 119) was significantly increased compared with the control group during the remission period, suggesting parasympathetic dysregulation in patients with CM. Our study showed that the time to maximum gradient recovery of the CM group at 2 min and 5 min after cold pressing was significantly longer than that of the HC group (*P* < 0.05). This is likely due to delayed pupillary sphincter withdrawal that is driven by increased parasympathetic tension. These findings suggest that patients with CM may have parasympathetic dysfunction, and that both CM and EM may be associated with ANS dysfunction.

The CPT is an experimental cold stimulus widely used to induce systemic sympathetic activation (Eren et al., 2018; Gazerani and Cairns, 2018). The stress response to cold stimulation is associated with the activation of two systems, the hypothalamic-pituitary-adrenal system and the sympathetic adrenomedullary system. When the stimulus is strong enough, it temporarily disturbs the homeostasis of the body, and the sympathetic adrenomedullary system triggers the ANS to enhance sympathetic nerve activity. In healthy subjects, the parasympathetic system is subsequently activated to maintain the homeostasis of the body (Gazerani and Cairns, 2018). The brain’s response to frequent or severe stressors may change, and can manifest as atypical behavioral and physiological impairments. Patients with CM suffer from persistent headaches, which may alter the function and structure of the brain, and which impair the homeostasis of the body. When the loading time of cold stimulation increases, the ANS is in a state of imbalance and the parasympathetic nerves are dysregulated. Delayed parasympathetic withdrawal resulted in a prolonged time to maximum gradient recovery at the initial stage of pupillary recovery. We also found that migraine attacks were induced within 24 h of cold stimulation in 40% of the CM cases, while the HC group did not experience any discomfort (e.g., headache). These results indicated that patients with CM were unable to adapt to the same type of stressor due to an altered homeostasis (e.g., ANS dysfunction) and were unable to inhibit stress responses in a timely manner. This ultimately led to an enhanced compensatory response (e.g., central sensitization), as manifested by migraine attacks (Bremner, 2009; Pelletier et al., 2015).

In the present study, we found no significant difference in the parameters of the pupillary light reflex and cardiovascular parameters between the CM and HC groups at baseline, indicating that the ANS of patients with CM may not be significantly affected during the remission period. Also, there was no significant difference in the blood pressure and heart rate at all time points of the CPT between the two groups. This suggested that autonomic innervation of the cardiovascular system in patients with CM may not be impaired by continuous sympathetic activation during the CPT, which is consistent with the findings of Benarroch (1993) and Mamontov et al. (2016). In the study by Eren et al. (2018), patients with EM showed normal autonomic innervation of the cardiovascular system after the CPT. Taken together, we speculate that autonomic innervation of the cardiovascular system in patients with CM may not be different from that in patients with EM even though attacks in CM are more frequent relative to EM.

**FIGURE 6:**
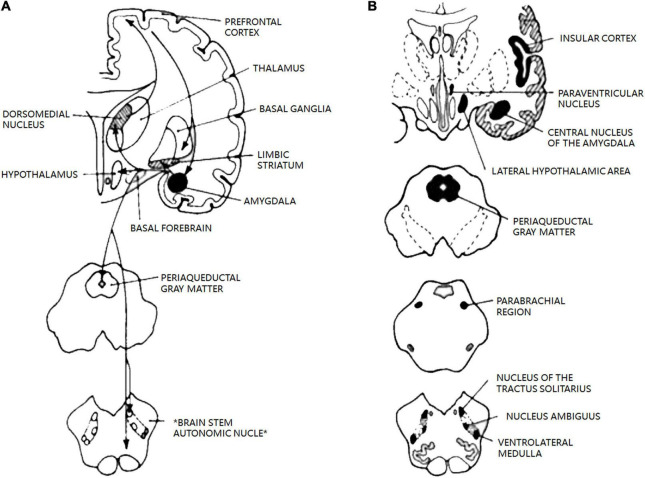
The central autonomic network. Reprinted with permission from Benarroch et al. The central autonomic network: functional organization, dysfunction, and perspective. Mayo Clin Proc [Internet].1993 Oct. **(A)** Diagram of main connections between central nucleus of amygdala and areas involved in autonomic and behavioral responses associated with emotion. **(B)** Schematic diagram of the most important areas of the central autonomic network and their location in human brain. Allareas are reciprocally interconnected; pathways have been omitted for clarit.

The ANS is a diffuse network that controls almost all unconscious processes in the body and is intrinsically involved in the regulation of physiological pain responses, including migraine. The central autonomic control center includes the central autonomic network, an interconnected nuclear system in the cortex and brainstem that regulates visceromotor, neuroendocrine, respiration, and pain responses (Figure 6; Borsook et al., 2012). The central autonomic network is thought to be involved in the pathogenesis of migraine, including alterations in the periaqueductal gray matter, parabrachial, dorsal nucleus of the vagus nerve (DMV), and ventrolateral medulla (Benarroch, 1993).

In this study, we used rs-fMRI to analyze 19 ANS-related brain areas and found that the ReHo values of the left lateral hypothalamic area and left amygdala of patients with CM were significantly increased compared with those of the HC group, indicating functional abnormalities in these brain areas in CM. This is the first time that the alterations in the amygdala have been detected by rs-fMRI in patients with migraine. In addition, no significant difference was found in the DTI indices of the 19 brain areas or the results of TBSS analysis of white matter skeleton between the two groups. This indicates that the white matter structure of ANS-related brain areas did not change significantly in patients with CM, which is consistent with previous study by Planchuelo-Gómez et al. (2020). They found that patients with chronic migraine may have chronic migraine in the first few months of the condition compared with those with episodic migraine. The axon integrity of patients with chronic migraine was impaired but the axons appeared to be normal at a later stage, suggesting the occurrence of a series of remodeling changes in the white matter of these patients. These findings were consistent with ours. The average disease course of our patients was more than 10 years and the results of TBSS were not significantly different between the CM and HC groups. In our future studies, seed point-based functional connection analysis will be performed to investigate functional connections among different abnormal brain regions and between abnormal brain regions and the rest of the whole brain. The region of interest (ROI) (autonomous nerve-related region) will be selected, and the fMRI time series of the region will be extracted and compared with that of the whole brain.

The advantages of this study are as follows: (1) This work was a controlled trial with large sample size; (2) The pathological mechanisms of ANS dysfunction in patients with CM were investigated using CPT, rs-fMRI, and DTI. (3) This study showed for the first time that the ReHo value of the amygdala in patients with CM was increased, providing clues to the central pathological mechanisms of autonomic symptoms in these patients. Concerning the limitations, a large number of patients with CM (80%) were found to have drug abuse, which might be a confounding factor. Also, we did not distinguish migraine with aura from migraine without aura, which might lead to a deviation of the current results.

In conclusion, patients with CM exhibited parasympathetic dysfunction and showed increased ReHo values in the amygdala. These results may provide clues to the pathogenic mechanisms of ANS dysfunction in patients with CM.

## Data Availability Statement

The raw data supporting the conclusions of this article will be made available by the authors, without undue reservation.

## Ethics Statement

The studies involving human participants were reviewed and approved by Medical Ethics Committee of the Affiliated Hospital of Chengdu University of TCM. The patients/participants provided their written informed consent to participate in this study.

## Author Contributions

MS: data curation, investigation, project administration, resources, supervision, validation, visualization, writing—original draft, and writing—review and editing. DL: data curation, validation, and writing—review and editing. JG: supervision, visualization, and writing—original draft. DY: writing—review and editing. ZL: data curation. HZ: data curation, investigation, resources, validation, visualization, writing-original draft, and writing—review and editing. All authors read and approved the final manuscript.

## Conflict of Interest

The authors declare that the research was conducted in the absence of any commercial or financial relationships that could be construed as a potential conflict of interest.

## Publisher’s Note

All claims expressed in this article are solely those of the authors and do not necessarily represent those of their affiliated organizations, or those of the publisher, the editors and the reviewers. Any product that may be evaluated in this article, or claim that may be made by its manufacturer, is not guaranteed or endorsed by the publisher.

## Footnotes

1
www.virtualbox.org

2
https://afni.nimh.nih.gov/pub/dist/doc/manual/AlphaSim.pdf

3
http://rfmri.org/dpabi

4
http://www.alivelearn.net/xjview

## Abbreviations

ANS: autonomic nervous system
CM: chronic migraine
DTI: diffusion tensor imaging
ReHo: regional homogeneity
VIP: vasoactive intestinal peptide
CPT: cold pressor test
fMRI: functional magnetic resonance imaging
TCM: traditional Chinese Medicine
HIS: international Headache Society
NSAIDs: non-steroidal anti-inflammatory drugs
VAS: visual analogue scale
HC: healthy control
FOV: field-of-view
TR: repetition time
TE: echo time
FA: flip angle
SBS: spacing between slices
EPI: echo-planar imaging
TBSS: tract based spatial statistics
FA: fractional Anisotropy
MD: mean diffusivity
RD: radial diffusivity
AD: axial diffusivity
rs-fMRI: resting-state functional magnetic resonance imaging.
